# Higher neutrophil to lymphocyte ratio is associated with renal dysfunction and cardiac adverse remodeling in elderly with metabolic syndrome

**DOI:** 10.3389/fcvm.2022.921204

**Published:** 2022-09-08

**Authors:** Yuqi Zhu, Gang Li, Jari A. Laukkanen, Xing Song, Jing Zhang, Linping Wei, Xinrui Chen, Yufeng Li, Cheng Liu

**Affiliations:** ^1^Division of Cardiology, Department of Geriatrics, The First Affiliated Hospital of Chongqing Medical University, Chongqing, China; ^2^Institute of Public Health and Clinical Nutrition, University of Eastern Finland, Kuopio, Finland; ^3^Department of Medicine, Institute of Clinical Medicine, University of Eastern Finland, Kuopio, Finland; ^4^Department of Medicine, Central Finland Health Care District, Jyväskylä, Finland

**Keywords:** neutrophil/lymphocyte ratio, inflammation, renal dysfunction, cardiac remodeling, metabolic syndrome, elderly, age, gender

## Abstract

**Background:**

Previous studies have shown that metabolic syndrome (MetS) is associated with increased systemic inflammation and cardiac mortality in elderly subjects. However, information on the association of inflammation markers with cardiac adverse remodeling is limited in the elderly with MetS. Therefore, we investigated whether the inflammatory marker neutrophil/lymphocyte ratio (NLR) is associated with the cardiac adverse remodeling in Chinese elderly with MetS.

**Methods:**

A total of 1,087 hospitalized Chinese elderly (aged ≥ 65 years) with MetS were collected retrospectively. The cross-sectional data of echocardiography and clinical parameters were compared among quartile NLR groups.

**Results:**

In the elderly with MetS, higher quartile NLR (≥3.83) was found to be associated with male gender, older age, lower estimated glomerular filtration rate (eGFR), and cardiac left ventricular (LV) dilatation (all *p* <0.05).

**Conclusion:**

Higher NLR is associated with male gender, older age, renal dysfunction, and cardiac adverse remodeling in Chinese elderly with MetS.

## Introduction

Metabolic syndrome (MetS) presents with a cluster of chronic metabolic abnormalities, which include obesity, hyperglycemia, high blood pressure (BP) hypertriglyceridemia, and decreased high-density lipoprotein cholesterol (HDL-C) (1). MetS is considered to be a disorder with chronic systemic low-grade inflammation (2). The inflammatory response plays a central role in the development and progression of MetS and its unwanted consequences, such as atherosclerotic cardiovascular diseases (3). Recent studies reported that neutrophil to lymphocyte ratio (NLR), as a marker of inflammation, was increased in the MetS. Indeed, NLR could be used as one potential marker of MetS development (4, 5). Furthermore, higher NLR was found to be associated with the incidence of the MetS components, such as hypertension and type 2 diabetes mellitus (DM) (6–8). Previous studies have demonstrated an association between higher NLR and the severity of coronary artery disease (9, 10). Moreover, NLR was reported to be a strong prognostic indicator for cardiac adverse remodeling (11), an increased risk of cardiac events, and mortality from coronary artery disease (12–15). NLR is a promising biomarker, which is an easily available measure in clinical practice. NLR measurement has a high effectiveness-cost ratio and good reliability due to its lower variability by treatments (5, 12, 16). In addition, MetS is a common clinical condition in the elderly people (17–19). Studies have shown that elderly patients with MetS were vulnerable to increased coronary atherosclerosis-related events and cardiac mortality (20–23). MetS has been found to be related to cardiac structural changes and left ventricular (LV) dysfunction in adults (24, 25). A higher level of inflammation and increased incidence of atherosclerosis-associated LV dysfunction have been discovered in the elderly with MetS (26). However, there is still limited information on whether NLR is correlated with the adverse cardiac structural remodeling in the elderly with MetS. Therefore, the aim of this study was to explore whether NLR is associated with the cardiac adverse remodeling in Chinese elderly with MetS.

## Methods

### Patients

At the department of endocrinology in our hospital, a total of 1,087 Chinese elderly (aged ≥ 65 years) with MetS were collected retrospectively by review of medical records from 2014 to 2020 in a cross-sectional study.

In the present study, MetS was defined as having three or more of the following abnormalities (27). (1) Fasting plasma glucose (PG) impairment (7 mmol/L > fasting PG ≥ 6.1 mmol/L), oral 75 g glucose post-loaded 2-h PG (2 hPG) impairment (11.1 mmol/L > 2 hPG ≥ 7.8 mmol/L), or confirmed type 2 DM that is under anti-diabetic treatments. Type 2 DM was diagnosed if fasting PG ≥7.0 mmol/L and/or 2 hPG ≥11.1 mmol/L, while the subject's plasma insulin level was normal or increased (28). (2) High normal BP (140 mmHg > systolic BP ≥ 130 and/or 90 mmHg > diastolic BP ≥ 85 mmHg) or hypertension with the use of medication. Hypertension was confirmed if the subjects had average systolic BP ≥ 140 mmHg and/or diastolic BP ≥90 mmHg in upper right arm after 10 min of rest and two measurements of BP. (3) Hypertriglyceridemia [fasting plasma triglyceride (TG) ≥ 1.70 mmol/L]. (4) Decreased HDL-C (fasting plasma HDL-C <1.03 mmol/L in men; HDL-C <1.29 mmol/L in women). The MetS criterion used in this study was from the National Cholesterol Education Program Adult Treatment Panel III (27). However, in our study, the patient's information on waist circumference was not available from the medical records. Thus, the waist circumference was not used in the diagnosis of MetS in this study.

Gout, which was triggered by the crystallization of monosodium urate monohydrate inside the joints, was considered when there were gouty tophus, arthralgia, and arthritis (29). Coronary heart disease was diagnosed if there were signs of myocardial infarction on 12-lead electrocardiograms (ECG), angiography showed atherosclerotic stenosis ≥50% in one of the coronary artery lumens, or a history of coronary stent implantation was confirmed. Stroke criteria were based on typical findings on computed tomograms or magnetic resonance imaging (MRI) (30). All patients were examined by color Doppler echocardiography. White blood cell (WBC) count was assessed using a Sysmex XN-1000 Analyzer (Sysmex Corporation, Kobe, Japan). The NLR was then calculated manually. The WBC and NLR were measured averagely of three times within the first week of hospitalization for each patient, respectively.

We excluded patients with the infection that included acute viral illness, autoimmune diseases, acute gout, stroke, myocardial infarction, rheumatic or degenerative cardiac valvular disease, congenital heart disease, idiopathic cardiomyopathy, atrial fibrillation, chronic obstructive pulmonary disease, leukemia, and other malignant tumors.

### Calculation of glomerular filtration rate and body mass index

For men, serum creatinine (Scr) > 80 μmol/L: estimated GFR [eGFR = 141 (Scr μmol/L/88.4/0.9)^−1.209^ 0.993^age(years)^]; Scr ≤ 80 μmol/L: [eGFR = 141 (Scr μmol/L/88.4/0.9)^−0.411^ · 0.993^age(years)^]. For women, Scr > 62 μmol/L, eGFR = 144 × (Scr μmol/L/88.4/0.7) ^−1.209^ × 0.993^age(years)^; Scr ≤ 62 μmol/L, eGFR = 144 × (Scr μmol/L/88.4/0.7) ^−0.329^ × 0.993^age(years)^ (31). BMI = body weight (kg)/[height (m)]^2^ (32, 33).

### Determination of clinical biochemistry

Glucose was measured by hexose enzyme colorimetry. Hemoglobin A1c (HbA1c) was determined by high-performance liquid chromatography. TG and total cholesterol were detected by enzyme colorimetry. HDL-C was measured by homogeneous enzyme colorimetry. Low-density lipoprotein cholesterol (LDL-C) was calculated by the Friedewald formula. High sensitivity C-reactive protein (hsCRP) was determined by immunoturbidimetry (34).

### Measurement of cardiac structure and function

Right atrial diameter, right ventricular diameter (RVD), left atrial diameter (LAD), LV end-systolic (LVESD) and end-diastolic diameter (LVEDD), interventricular septal thickness (IVST), LV posterior wall thickness (LVPWT), and LV ejection fraction (LVEF) were measured by transthoracic two-dimensional M-mode echocardiography using GE Vivid7 with full digital color Doppler ultrasound diagnostic instrument (35).

### Statistical analysis

The statistical package for social science (SPSS) 26.0 software (IBM Company, Chicago, IL, USA) was used for statistical analysis. The cutoff values of quartile NLR used in the current study were calculated by the SPSS software, according to the NLR values of the elderly subjects in the present study. The categorical data were expressed as a percentage (%) and compared by chi-square test between two groups or Fisher exact test between multiple groups. The continuous data were expressed as mean values ± standard deviation (SD). If the variables were normally distributed, they were analyzed by one-way analysis of variance (ANOVA) between multiple groups, then followed by *post-hoc* analysis for comparison between two groups by least significance difference (LSD) test. If the data were in non-normal distribution, it was analyzed by Kruskal–Wallis *H* test between multiple groups. Then, the Mann–Whitney *U* test was used for comparison between the two groups. Pearson correlation analysis was used for univariate analysis. Dichotomized logistic regression analysis was used for multivariate analysis. Given that WBC, neutrophil count, lymphocyte count, and hsCRP were collinear to NLR, these parameters were not included in the regression analysis. A 2-tailed value of *p* < 0.05 was considered to be statistically significant.

## Results

### Clinical characteristics corresponding to quartile NLR

The clinical characteristics of subjects according to the quartile of NLR are shown in Table 1. Compared with the first quartile NLR (NLR <2.03) group, patient's prevalence rates of male gender, HbA1c, WBC, neutrophil count, and hsCRP were higher; while eGFR and lymphocyte count were lower in the second quartile NLR (2.03 ≤ NLR <2.82) group (All *p* < 0.05, Table 1). Prevalence rates of male gender and smoking, HbA1c, WBC, neutrophil count, and hsCRP were higher; diabetes and hypertension had a longer duration; while TG, HDL-C, eGFR, and lymphocyte count were lower in the second quartile NLR (2.03 ≤ NLR <2.82) group (All *p* < 0.05, Table 1). Prevalence rates of male gender and smoking, HbA1c, WBC, neutrophil count, and hsCRP were higher; diabetes and hypertension had a longer duration; while TG, HDL-C, eGFR, and lymphocyte count were lower in the third quartile NLR (2.82 ≤ NLR <3.83) group (All *p* < 0.05, Table 1); prevalence rates of male gender, smoking, drinking and gout, systolic and diastolic BP, HbA1c, WBC, neutrophil count, and hsCRP were higher; age was older; diabetes, hypertension, and gout had a longer duration; while TG, HDL-C, LDL-C, eGFR, and lymphocyte count were lower in the fourth quartile NLR (NLR ≥ 3.83) group (All *p* < 0.05, Table 1).

**Table 1:** Comparison of clinical characteristics among quartile NLR groups.

	**1st quartile**	**2nd quartile**	**3rd quartile**	**4th quartile**	***r***	***P-*value**
	**(NLR <2.03)**	**(2.03 ≤ NLR <2.82)**	**(2.82 ≤ NLR <3.83)**	**(NLR ≥3.83)**		
	**(*n* = 274)**	**(*n* = 269)**	**(*n* = 272)**	**(*n* = 272)**		
NLR	1.55 ± 0.31	2.43 ± 0.23^*^	3.26 ± 0.3^*^^†^	5.88 ± 2.54^*^^†^^‡^	1.000	<0.001
Male, *n* (%)	102 (37.2)	126 (46.8)^*^	155 (57.0)^*^^†^	185 (68.0)^*^^†^^‡^	0.230	<0.001
Age, years	72.59 ± 6.25	73.39 ± 6.42	73.67 ± 6.59	74.72 ± 6.58^*^^†^	0.087	0.004
Body mass index, kg/m^2^	25.20 ± 3.24	25.14 ± 2.93	25.09 ± 3.27	24.65 ± 3.47	−0.056	0.066
Smoking, *n* (%)	64 (23.4)	82 (30.5)	90 (33.1)^*^	124 (45.6)^*^^†^^‡^	0.165	<0.001
Drinking, *n* (%)	46 (16.8)	55 (20.4)	57 (21.0)	71 (26.1)^*^	0.078	0.010
Coronary heart disease, *n* (%)	73 (26.6)	87 (32.3)	93 (34.2)	93 (34.2)	0.059	0.052
Stroke, *n* (%)	63 (23.0)	58 (21.6)	52 (19.1)	66 (24.3)	0.004	0.905
Impaired PG, *n* (%)	6 (2.2)	4 (1.5)	3 (1.1)	2 (0.7)	−0.039	0.204
Type 2 DM, *n* (%)	259 (94.5)	260 (96.7)	265 (97.4)	265 (97.4)	0.058	0.056
Diabetic duration, years	10.74 ± 7.62	11.87 ± 8.64	12.34 ± 8.26^*^	12.72 ± 7.92^*^	0.089	0.003
High normal BP, *n* (%)	12 (4.4)	7 (2.6)	14 (5.1)	5 (1.8)	−0.031	0.306
Hypertension, *n* (%)	252 (92.0)	258 (95.9)	256 (94.1)	258 (94.9)	0.033	0.276
Hypertensive duration, years	9.87 ± 9.57	11.42 ± 10.97	12.17 ± 10.92^*^	12.48 ± 11.85^*^	0.060	0.048
Systolic BP, mmHg	141.43 ± 22.61	142.26 ± 18.93	143.23 ± 18.62	145.97 ± 18.84^*^^†^	0.065	0.042
Diastolic BP, mmHg	74.23 ± 13.44	74.62 ± 10.96	75.61 ± 11.96	76.70 ± 12.75^*^^†^	0.063	0.043
Gout, *n* (%)	10 (3.6)	15 (5.6)	19 (7.0)	31 (11.4)^*^^†^^‡^	0.096	0.002
Gout duration, years	0.31 ± 2.37	0.43 ± 2.61	0.71 ± 3.42	1.19 ± 4.53^*^^†^	0.080	0.008
HbA1c, %	8.15 ± 1.84	8.53 ± 2.26^*^	8.61 ± 2.21^*^	8.69 ± 2.25^*^	0.064	0.042
Triglyceride, mmol/L	2.44 ± 1.66	2.22 ± 1.33	2.17 ± 1.51^*^	2.05 ± 1.68^*^	−0.088	0.004
HDL-C, mmol/L	0.95 ± 0.2	0.98 ± 0.22	0.90 ± 0.23^*^^†^	0.90 ± 0.27^*^^†^	−0.101	0.001
LDL-C, mmol/L	2.48 ± 0.9	2.60 ± 0.97	2.34 ± 0.89^†^	2.26 ± 0.94^*^^†^	−0.112	<0.001
eGFR, mL/min/1.73 m^2^	74.14 ± 19.30	70.31 ± 22.37^*^	69.29 ± 21.26^*^	61.24 ± 25.43^*^^†^^‡^	−0.196	<0.001
White blood cell, ×10^9^/L	5.96 ± 1.81	6.71 ± 1.57^*^	6.6 ± 1.6^*^	7.19 ± 1.75^*^^†^^‡^	0.231	<0.001
Neutrophil, ×10^9^/L	3.19 ± 1.02	4.27 ± 1.03^*^	4.55 ± 1.07^*^^†^	5.52 ± 1.46^*^^†^^‡^	0.570	<0.001
Lymphocyte, ×10^9^/L	2.12 ± 0.85	1.77 ± 0.42^*^	1.40 ± 0.34^*^^†^	1.02 ± 0.32^*^^†^^‡^	−0.615	<0.001
hsCRP, mg/L	3.03 ± 5.00	4.25 ± 5.92^*^	5.37 ± 6.93^*^^†^	7.92 ± 7.99^*^^†^^‡^	0.261	<0.001

Values presented as mean ± SD or n (%).

* p <0.05 vs. 1st quartile NLR group;

† p <0.05 vs. 2nd quartile NLR group;

‡ p <0.05 vs. 3rd quartile NLR group; NLR, neutrophil/lymphocyte ratio; PG, plasma glucose; DM, diabetes mellitus; BP, blood pressure; HbA1c, glycated hemoglobin; HDL-C, high-density lipoprotein cholesterol; LDL-C, low-density lipoprotein cholesterol; eGFR, estimated glomerular filtration rate; hsCRP, high sensitivity C-reactive protein.

Compared with the second quartile NLR group, the prevalence rates of the male gender, neutrophil count, and hsCRP were higher; while HDL-C, LDL-C, and lymphocyte count were lower in the third quartile NLR group (All *p* < 0.05, Table 1); prevalence rates of the male gender, smoking and gout, systolic and diastolic BP, WBC, neutrophil count, and hsCRP were higher; age was older; gout had a longer duration; while HDL-C, LDL-C, eGFR, and lymphocyte count were lower in the fourth quartile NLR group (all *p* < 0.05, Table 1). Compared with the third quartile NLR group, prevalence rates of the male gender, smoking and gout, WBC, neutrophil count, and hsCRP were higher; while eGFR and lymphocyte count were lower in the fourth quartile NLR group (all *p* < 0.05, Table 1).

**Figure 1:**
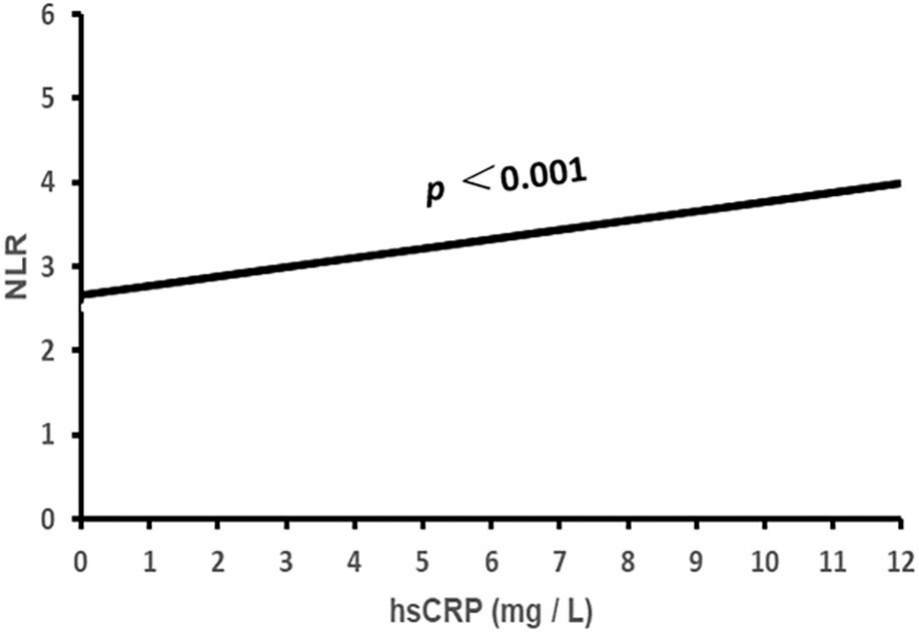
The association of NLR with hsCRP. NLR, neutrophil to lymphocyte ratio; hsCRP, high sensitivity C-reactive protein.

Binary correlation analysis showed that NLR was correlated positively with prevalence rates of male gender, smoking, drinking and gout, age, diabetes, hypertensive and gout duration, systolic and diastolic BP, HbA1C, WBC, neutrophil count, and hsCRP; while it was correlated reversely with TG, HDL-C, LDL-C, eGFR, and lymphocyte count (all *p* < 0.05, Table 1, Figure 1).

### Cardiac remodeling corresponding to quartile NLR

The results on the cardiac remodeling of subjects according to the quartile of NLR are shown in Table 2. Compared with first quartile NLR group, patient's LAD and LVEDD were larger in the second quartile NLR group (all *p* < 0.05, Table 2); RVD, LAD, LVESD, LVEDD, IVST, and LVPWT were larger in the third quartile NLR group (all *p* < 0.05, Table 2); and RVD, LAD, LVESD, LVEDD, IVST, and LVPWT were larger, while LVEF was lower in the fourth quartile NLR group (all *p* < 0.05, Table 2).

**Table 2:** Comparison of echocardiographic parameters among quartile NLR groups.

	**1st quartile**	**2nd quartile**	**3rd quartile**	**4th quartile**	***r***	***P-*value**
	**(NLR <2.03)**	**(2.03 ≤ NLR <2.82)**	**(2.82 ≤ NLR <3.83)**	**(NLR ≥3.83)**		
	**(*n* = 274)**	**(*n* = 269)**	**(*n* = 272)**	**(*n* = 272)**		
RAD, mm	32.50 ± 2.75	32.77 ± 2.98	32.84 ± 2.97	32.74 ± 3.47	0.055	0.073
RVD, mm	19.14 ± 1.79	19.34 ± 1.80	19.52 ± 1.55^*^	19.48 ± 2.04^*^	0.076	0.012
LAD, mm	30.16 ± 3.70	30.81 ± 3.86^*^	30.85 ± 3.49^*^	31.17 ± 4.05^*^	0.090	0.003
LVESD, mm	30.65 ± 3.92	31.03 ± 3.61	31.36 ± 3.74^*^	32.23 ± 4.27^*^^†^^‡^	0.126	<0.001
LVEDD, mm	46.50 ± 4.83	47.50 ± 4.67^*^	47.31 ± 4.57^*^	48.60 ± 4.80^*^^†^^‡^	0.144	<0.001
IVST, mm	10.94 ± 1.25	11.11 ± 1.28	11.35 ± 1.2^*^^†^	11.21 ± 1.27^*^	0.094	0.002
LVPWT, mm	10.73 ± 1.18	10.90 ± 1.12	11.04 ± 1.16^*^	10.98 ± 1.22^*^	0.087	0.004
LVEF, %	63.34 ± 4.49	63.35 ± 4.41	62.81 ± 5.03	62.26 ± 5.35^*^^†^	−0.062	0.041
E/A <1, *n* (%)	257 (93.8)	252 (93.7)	258 (94.9)	244 (89.7)	−0.049	0.109

Values presented as mean ± SD.

* p <0.05 vs. 1st quartile NLR group;

† p <0.05 vs. 2nd quartile NLR group;

‡ p <0.05 vs. 3rd quartile NLR group; NLR, neutrophil/lymphocyte ratio; RAD, right atrial diameter; RVD, right ventricular diameter; LAD, left atrial diameter; LVESD, left ventricular end-systolic diameter; LVEDD, left ventricular end-diastolic diameter; IVST, interventricular septal thickness; LVPWT, left ventricular posterior wall thickness; LVEF, left ventricular ejection fraction; E/A, peak early (E)/late (A) filling velocities.

Compared with the second quartile NLR group, IVST was larger in the third quartile NLR group (all *p* < 0.05, Table 2); LVESD and LVEDD were larger, while LVEF was lower in the fourth quartile NLR group (all *p* < 0.05, Table 2); and compared with third quartile NLR group, LVESD and LVEDD were larger in the fourth quartile NLR group (all *p* < 0.05, Table 2).

Binary correlation analysis showed that NLR was correlated positively with RVD, LAD, LVESD LVEDD, IVST, and LVPWT, while it was correlated reversely with LVEF (all *p* < 0.05, Table 2).

### Correlation of NLR with clinical characteristics and cardiac remodeling

**Figure 2:**
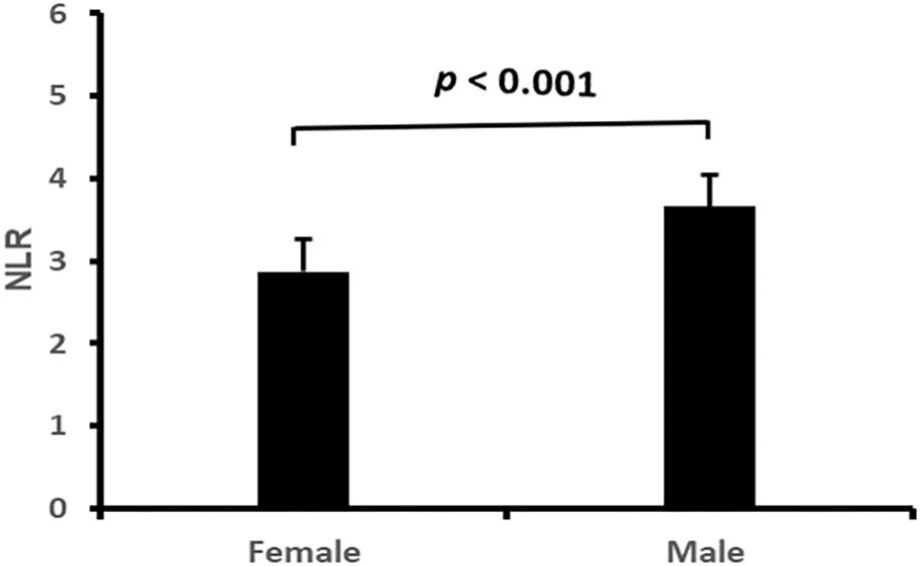
The NLR in man and woman. NLR, neutrophil to lymphocyte ratio.

**Figure 3:**
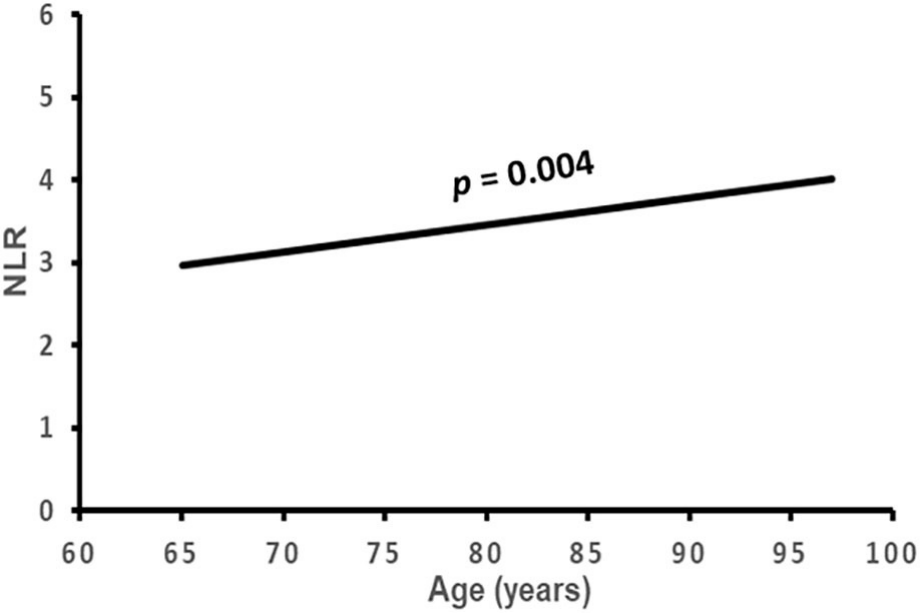
The association of NLR with age. NLR, neutrophil to lymphocyte ratio.

**Figure 4:**
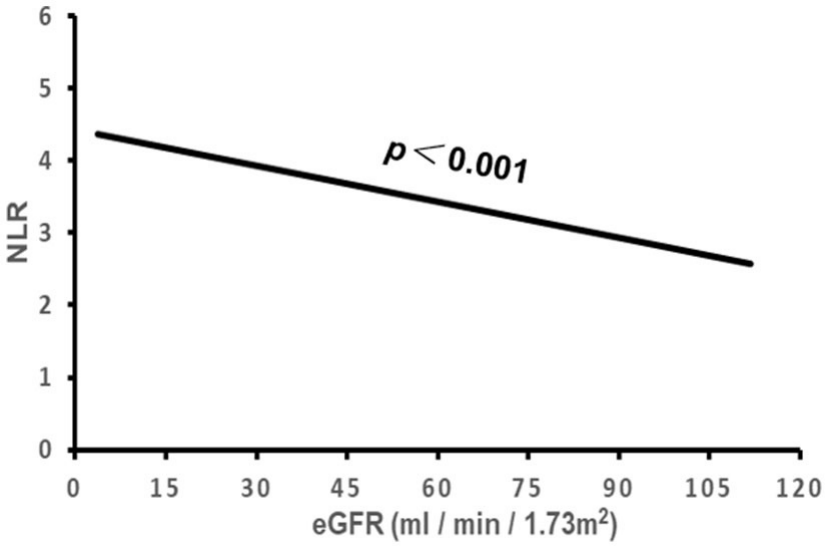
The association of NLR with eGFR. NLR, neutrophil to lymphocyte ratio; eGFR, estimated glomerular filtration rate.

**Figure 5:**
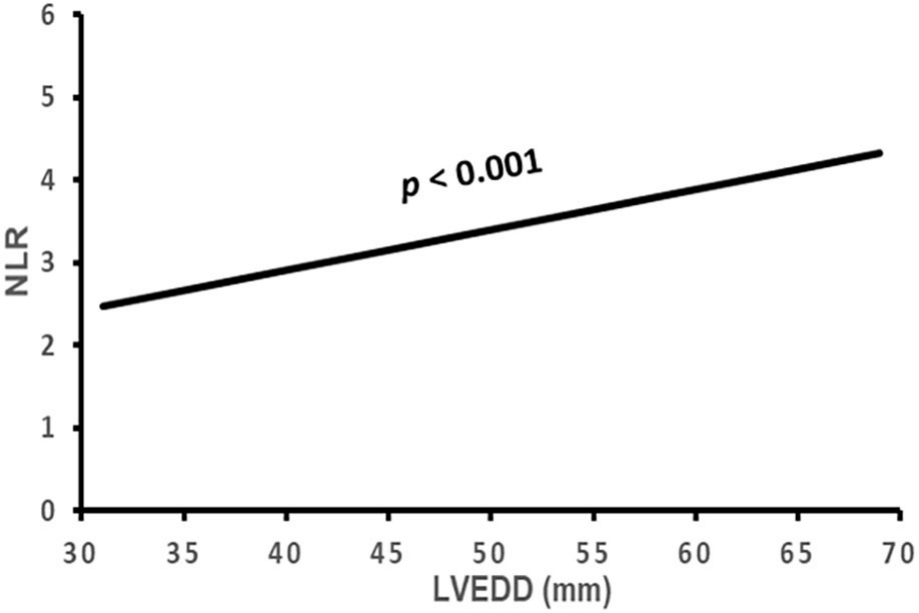
The association of NLR with LVEDD. NLR, neutrophil to lymphocyte ratio; LVEDD, left ventricular end-diastolic diameter.

The correlation of NLR with the subject's clinical characteristics and cardiac remodeling is shown in Table 3 and Figures 2–5. Dichotomized variable regression analysis showed that the fourth quartile NLR (≥3.83) was associated independently with male gender, older age, decreased eGFR, and cardiac LV dilatation (all *p* < 0.05, Table 3, Figures 2–5), after adjustment of patient's other parameters.

**Table 3:** Regression analysis of parameters associated with NLR ≥ 3.83.

	**β**	**SE**	**Wald**	***P*-value**	**OR (95% CI)**
Male	0.629	0.219	8.265	0.004	1.876 (1.222–2.881)
Age	0.026	0.013	4.158	0.041	1.026 (1.001–1.052)
Smoking	0.358	0.213	2.810	0.094	1.430 (0.941–2.173)
Drinking	−0.119	0.204	0.341	0.559	0.888 (0.595–1.324)
Diabetic duration	0.010	0.010	0.955	0.329	1.010 (0.990–1.030)
Hypertensive duration	−0.014	0.008	3.461	0.063	0.986 (0.971–1.001)
Systolic BP	−0.002	0.005	0.229	0.632	0.998 (0.989–1.007)
Diastolic BP	0.000	0.008	0.001	0.972	1.000 (0.985–1.016)
Gout	0.120	0.406	0.087	0.768	1.127 (0.508–2.500)
Gout duration	0.008	0.029	0.077	0.782	1.008 (0.952–1.068)
HbA1c	−0.007	0.036	0.037	0.847	0.993 (0.925–1.066)
Triglyceride	−0.073	0.056	1.740	0.187	0.929 (0.833–1.036)
HDL-C	−0.175	0.361	0.235	0.628	0.840 (0.414–1.703)
LDL-C	−0.164	0.094	3.026	0.082	0.849 (0.706–1.021)
eGFR	−0.018	0.004	24.359	<0.001	0.983 (0.976–0.989)
RVD	−0.009	0.045	0.044	0.833	0.991 (0.907–1.082)
LAD	0.003	0.024	0.020	0.888	1.003 (0.957–1.052)
LVESD	−0.169	0.105	2.583	0.108	0.845 (0.688–1.038)
LVEDD	0.139	0.070	3.973	0.046	1.149 (1.002–1.317)
IVST	−0.012	0.101	0.013	0.908	0.988 (0.810–1.206)
LVPWT	0.000	0.107	0.000	1.000	1.000 (0.811–1.234)
LVEF	−0.063	0.039	2.660	0.103	0.939 (0.870–1.013)

NLR, neutrophil/lymphocyte ratio; β, regression coefficient; SE, standard error; Wald, Chi square value; CI, confidence interval; OR, odds ratio; BP, blood pressure; HbA1c, glycated hemoglobin; HDL-C, high-density lipoprotein cholesterol; LDL-C, low-density lipoprotein cholesterol; eGFR, estimated glomerular filtration rate; RVD, right ventricular diameter; LAD, left trial diameter; LVESD, left ventricular end-systolic diameter; LVEDD, left ventricular end-diastolic diameter; IVST, interventricular septal thickness; LVPWT, left ventricular posterior wall thickness; LVEF, left ventricular ejection fraction.

## Discussion

The present study shows that higher NLR is independently associated with male gender, older age, lower eGFR, and cardiac LV remodeling in Chinese elderly with MetS.

There are some potential underlying mechanisms explaining the association between NLR and cardiac LV remodeling. As a core component of the MetS, obesity usually manifests as increased fat accumulation in visceral organs. Moreover, abdominal obesity is commonly observed in patients with MetS (36). Obesity is often associated with dyslipidemia. The prevailing consensus is that the pro-inflammatory molecules mainly derive from excessive visceral adipose tissues in patients with MetS (37). The visceral adipose tissue can secrete pro-inflammatory adipokines, leading to local and systemic inflammatory responses (38). This phenomenon is called lipotoxicity (39). With unhealthy lifestyles and increase in age, fat accumulates more in viscera, leading to increased systemic inflammatory responses (2). In the present study, we found that older age was associated with higher inflammatory marker NLR in elderly MetS. This finding is consistent with other earlier results in the elderly population (40). Our study also found that higher NLR was more commonly seen in elderly men with MetS. A higher level of inflammation in the older men may be due to unhealthy lifestyles and older age (41, 42).

Excessive adipose tissue and its secreted pro-inflammatory mediators (37) may contribute mainly to the over activation of sympathetic nervous and renin angiotensin systems (RAS) in MetS (43). This activated RAS could also be involved interactively in the aggravation of inflammatory reactions (44). Then, the higher inflammatory responses lead to increased apoptosis in glomerular endothelial cells and ensuing chronic kidney insufficiency in MetS (45). Due to adipose tissue retention-associated insulin resistance and impaired use of glucose exist in MetS, all kinds of cells are forced to use more fatty acid oxidation for their energy supply (46). However, this kind of energy supply will inevitably result in an increase in cellular oxidative stresses and inflammatory responses. Meanwhile, type 2 DM and diabetic nephropathy appear consequently (47, 48). Long-term increased inflammatory responses eventually cause glomerular endothelial injury and chronic kidney disease (49). Higher inflammatory marker NLR was observed to be associated with renal dysfunction and poor renal outcomes in adults with chronic kidney disease (50, 51). However, to our knowledge, the present study is the first to show that higher NLR is associated with lower eGFR in the elderly with MetS.

As the similar underlying mechanisms mentioned above (37), the over activated sympathetic nerve and RAS in MetS (43, 44) may also lead to increased aldosterone, water-sodium retention, cardiac overload, higher BP, resultant increased cardiomyocytic apoptosis, and myocardial fibrosis. Subsequently, this may cause cardiac remodeling and dysfunction in MetS (52, 53). Indeed, the interactive effects between aldosterone and sympathetic nerve-RAS (54) may also contribute to cardiac remodeling and dysfunction. Furthermore, as the cellular insulin resistance and impaired use of glucose exist in MetS (46), excessive cellular oxidative stresses and inflammatory responses appear sequentially in MetS (47, 48). Chronic inflammatory responses in MetS can also cause cardiomyocytic injury and cardiac adverse remodeling (55). To our knowledge, the present study is the first to show that higher inflammatory marker NLR is associated with cardiac LV remodeling in the elderly with MetS.

## Limitations

There were no available data on the abdominal circumference, and we only used BMI to assess body weight and did not find the association between NLR and BMI in elderly MetS. However, BMI may not accurately reflect abdominal obesity. In addition, the medical records could not include all possible obesity assessments, such as abdominal circumference, which is an indicator for MetS diagnostic criteria. The abovementioned definition of gout did not include hyperuricemia. Gout and hyperuricemia are two different entities with varying clinicopathological implications. However, we did not differentiate hyperuricemia from gout in the patient data collection. The patients diagnosed with gout were required to have hyperuricemia in this study. Therefore, the reported prevalence of gout may not reflect the exact level of gout prevalence in this population, which is a study limitation. Furthermore, the current data were based on a cross-sectional study setting. Although multivariable regression analyses were performed, there could still be some other confounding factors that contributed to the observed associations. Therefore, the results of this study still need to be further verified by prospective randomized controlled trials in the future.

## Conclusion

Higher NLR is independently associated with male gender, older age, lower eGFR, and LV cardiac adverse remodeling in Chinese elderly with MetS. NLR may be used as a potentially cost-effective biomarker for renal and cardiac complications in MetS.

## Data availability statement

The original contributions presented in the study are included in the article/supplementary material, further inquiries can be directed to the corresponding author.

## Ethics statement

The studies involving human participants were reviewed and approved by The First Affiliated Hospital of Chongqing Medical University. Written informed consent for participation was not required for this study in accordance with the national legislation and the institutional requirements.

## Author contributions

GL contributed to the conception and design of the study. YZ organized the database. YZ, GL, XS, JZ, and CL performed the statistical analysis. YZ and GL wrote the first draft of the manuscript. JL, LW, XC, and YL: wrote sections of the manuscript. All authors contributed to manuscript revision, read, and approved the submitted version.

## Funding

This study was supported by the Natural Science Research Fund of the Medical Science and Technology Research Fund of Health Bureau of Chongqing City, China (Nos. 04-2-154 and 2009-2-290) and Chongqing Science and Technology Commission in Chongqing City, China (CSTC, No. 2007BB5276).

## Conflict of interest

The authors declare that the research was conducted in the absence of any commercial or financial relationships that could be construed as a potential conflict of interest.

## Publisher's note

All claims expressed in this article are solely those of the authors and do not necessarily represent those of their affiliated organizations, or those of the publisher, the editors and the reviewers. Any product that may be evaluated in this article, or claim that may be made by its manufacturer, is not guaranteed or endorsed by the publisher.
